# Localisation vaginale de Mansonella perstans: à propos d’un cas au centre hospitalier universitaire de Bobo-Dioulasso, Burkina Faso

**Published:** 2012-06-24

**Authors:** Sanata Bamba, Fatou Barro-Traoré, Martine Liance, Cathy Chemla, Charles Sanou, Olo Da, Tinga Robert Guiguemdé

**Affiliations:** 1 Service de Parasitologie –Mycologie du Centre Hospitalier Universitaire Sanou Souro, Bobo Dioulasso, Burkina Faso; 2 Service de Parasitologie -Mycologie de l’institut supérieur des sciences de la santé, Burkina Faso; 3 Service de Dermatologie du Centre Hospitalier Universitaire Yalgado Ouédraogo, Ouagadougou, Burkina Faso; 4 Service de Parasitologie et Mycologie, Centre Hospitalier Universitaire Henri Mondor, Créteil, France; 5 Laboratoire de Parasitologie-Mycologie, Hôpital Maison Blanche, Reims, France, Université polytechnique de Bobo Dioulasso, Burkina Faso

**Keywords:** Filarioses, Mansonella perstans, Frottis cervico-vaginal, Papanicolaou, Burkina Faso

## Abstract

Mansonella perstans est une filaire dont les adultes sont à localisation péritonéale et les microfilaires à localisation sanguine, qui sévit principalement en Amérique Equatoriale et aux bords de rivières, de plages en Afrique tropicale humide. Sa transmission est assurée par la piqûre de culicoïdes. Nous rapportons le cas d’une patiente souffrant de prurit dans un contexte biologique d’hyperéosinophilie au cours d’un dépistage du cancer du col de l’utérus. Une microfilaire de Mansonella perstans a été observée sur son frottis cervico-vaginal mais aussi dans son sang. La patiente a été traitée avec succès par une prise unique combinée de 400 mg d’albendazole et d’ivermectine (150 µg/kg). La localisation cervico-vaginale de Mansonella perstans est atypique et exceptionnelle. Nous proposons une recherche systématique de microfilaires lors de frottis cervico-vaginaux des femmes souhaitant un dépistage du cancer du col de l’utérus au Centre Hospitalier de Bobo-Dioulasso pour déterminer la fréquence réelle de cette localisation atypique.

## Introduction

Quatre espèces de filaires appartenant au genre Mansonella sont resFponsables des mansonelloses humaines. Trois d’entre elles sévissent en Afrique: *Mansonella perstans*, *M. streptocerca*, *M. ozzardi* et *M. rodhaini* [1–4]. Leur transmission est assurée par la piqûre de diptères hématophages qui abondent dans les écosystèmes tropicaux: les culicoïdes. Les adultes sont des vers de 35 à 80 mm de long [5]. Les femelles sont vivipares et les microfilaires circulent dans le sang périphérique sans périodicité nuit et jour. La longévité de la filaire adulte de *Mansonella perstans* chez l’homme est inconnue, celle des microfilaires est d’environ 4 mois [6]. Considérée comme peu ou pas pathogène (caractère pathogène occasionnel), cette filariose pourrait, cependant, être responsable d’arthralgies, de myalgies, d’éruptions cutanées, de fièvre et de troubles neurologiques [1, 7–9]. Comme la plupart des mansonelloses, celle à *Mansonella perstans* réalise une infection très tenace du fait de son insensibilité aux médicaments filaricides [10].

Nous rapportons un cas de mansonellose à *Mansonella perstans*, de localisation atypique, mis en évidence par l’examen d’un frottis cervico-vaginal chez une femme, au centre hospitalier universitaire de Bobo-Dioulasso (Burkina Faso).

## Patient et observation

Il s’agit d’une femme de 45 ans, originaire de la région de Mangodara, un village situé au sud de Bobo Dioulasso. Elle n’a jamais quitté son village. Elle a été admise pour un dépistage volontaire du cancer du col de l’utérus dans le service de gynécologie-obstétrique du Centre Hospitalier Universitaire Sourô Sanou (CHUSS) le 21 septembre 2010.

**Figure 1:**
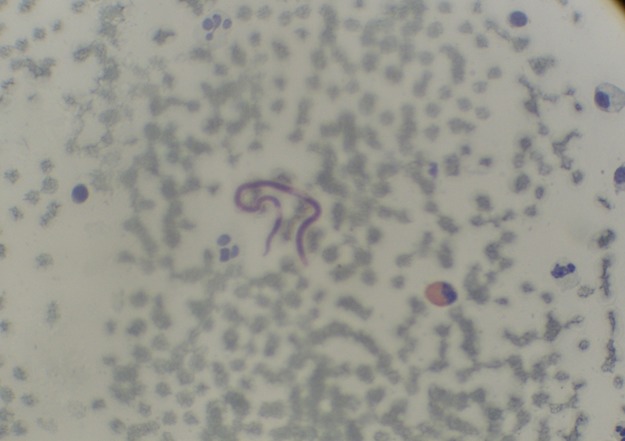
Microfilaire de Mansonella perstans sur frottis cervico-vaginal (Papanicolaou × 40)

A l’admission, l’examen clinique était normal mais nous avons noté un prurit cutané, isolé, modéré, épisodique, à prédominance nocturne, n’inquiétant pas la patiente. Avec un spéculum vaginal, un prélèvement du col de l’utérus a été réalisé avec une spatule plate pour la confection d’un frottis cervico-vaginal. Aucune lésion vaginale ou cervicale n’a été objectivée lors de cet examen. L′étalement et la fixation des cellules sur une lame porte-objet ont été réalisés. Dans le laboratoire de cytologie du CHUSS, la coloration de Papanicolaou [11] a été utilisée pour rechercher d’éventuelles cellules exfoliées sur le frottis. L’analyse cytologique du frottis coloré, n’a pas montré de lésions malpighiennes intra-épithéliales, ou de signe de malignité. Par contre, il a été notifié des modifications cellulaires réactionnelles, associées à une inflammation avec présence de polynucléaires éosinophiles et d’une microfilaire, de petite taille. La longueur de cette microfilaire a été estiméeà 180 µm et sa largeur à 5 µm. Elle était caractérisée par un espace céphalique court (3 µm). Ces noyaux somatiques étaient nombreux, ovoïdes, petits, à contours irréguliers et se chevauchant. L’extrémité postérieure de la microfilaire observée était rectiligne, arrondie « en doigt de gant », avec un noyau terminal. Elle n’avait pas de gaine (Figure 1). Ces caractéristiques indiquent que cette microfilaire était celle de *M. perstans*, conformément à la description de Ho Thi Sang et Petithory [12].

L’hémogramme a révélé une anémie microcytaire hypochrome avec un taux d’hémoglobine de 10,4 g/100 ml et un VGM à 71 µ^3^. Il existait une hyperleucocytose à 9000 leucocytes/ mm^3^ avec 8% d’éosinophiles (720 cellules/mm^3^).

Un prélèvement de sang veineux a été effectué chez la patiente à la recherche de microfilaires sanguicoles. Un examen à l’état frais entre lame et lamelle, une goutte épaisse et un frottis sanguin colorés au May-Grünwald Giemsa (MGG), réalisés, étaient tous positifs.

**Figure 2:**
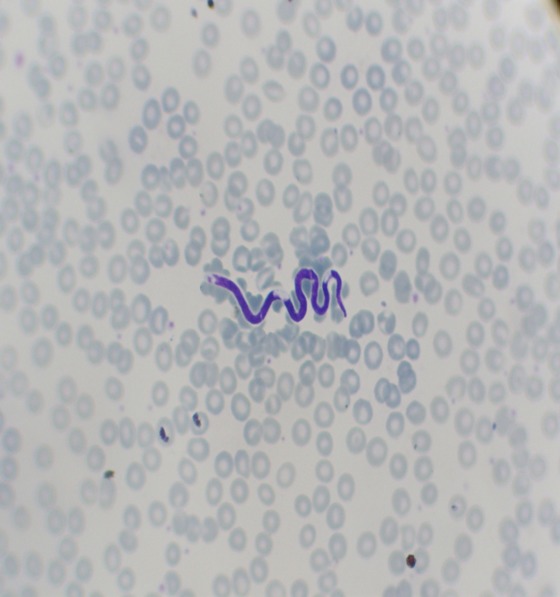
Microfilaire de Mansonella perstans sur un frottis sanguin (MGG × 40)

La densité parasitaire était toutefois inférieure à dix microfilaires/µl de sang. Des microfilaires, identiques à celles retrouvées sur le frottis cervico-vaginal, ont été identifiées dans le sang avec des noyaux somatiques de taille moyenne, irréguliers. Le dernier noyau était arrondi et avait l’aspect caractéristique dit « en doigt de gant ». Le corps interne n’était pas visible (Figure 2). Le reste du bilan biologique était normal.

La patiente a reçu par voie orale une prise unique combinée de 400 mg d’albendazole et d’ivermectine (150 µg/kg) ainsi que du chlorhydrate de prométhazine, à raison d’un comprimé à 25 mg renouvelable au bout de 4 heures. Trois mois après le traitement, la patiente ne se plaignait plus de prurit, elle avait un bon état général. Le bilan hématologique montrait une normalisation du taux des éosinophiles. Le frottis sanguin, l’examen à l’état frais entre lame et lamelle et la concentration du sang veineux par une solution de saponine selon la technique décrite par Ho Thi Sang et Petithory [12], étaient négatifs. Sur un nouveau prélèvement cervico-vaginal, coloré au MGG, aucune microfilaire n’a été observée.

## Discussion

La mansonellose à *Mansonella perstans* est la filariose humaine la plus représentée dans la région de Bobo Dioulasso. Elle y est plus fréquente que l’onchocercose et la wuchereriose [4]. Considérée comme non pathogène, elle pourrait être à l’origine de troubles cliniques variés avec, en particulier, des manifestations cutanées.

Sur le plan biologique, bon nombre d’auteurs s’accordent pour la considérer comme source d’une importante hyper éosinophilie sanguine [13, 14]. C’est ce qui justifie l’analyse du cas rapporté.

Chez cette patiente, le prurit était isolé, ne l’inquiétant pas; le frottis cervico-vaginal a permis de mettre en évidence, de façon fortuite, la microfilaire (Figure 1), ce qui nous a conduits à réaliser les autres examens complémentaires. Cette hyperéosinophilie a été confirmée chez cette patiente à la biologie et sur le frottis. Cependant, le siège vaginal des microfilaires n’est pas classique. Des cas isolés sont parfois signalés. C’est le cas de *Acanthocheilonema perstans* au Congo [15].

Seules deux études font état d’observations plus nombreuses de microfilaires dans les frottis cervico-vaginaux. Ainsi, Borges [16], à Caracas (Venezuela), a trouvé 12 cas de microfilaires (essentiellement O.volvulus) parmi 15261 frottis gynécologiques. Les microfilaires ont été volontiers associés à de nombreux neutrophiles ainsi qu’à des histiocytes et à du mucus. De même, Sharma [17] en Uganda, a signalé 243 cas de *Dipetolema perstans* parmi 40 000 frottis cervicaux. Des travaux réalisés au Gabon par Khalil et al [18] ont montré que des phénomènes inflammatoires, hémorragiques (menstruations, érosion du col de l’utérus, des polypes hémorragiques, métrorragie post–ménopause, et le cancer du col de l’utérus), la présence fréquente de bactéries et d’autres parasites dont les trichomonas (responsables d’atypies cellulaires diverses) sont pratiquement toujours associés à la localisation vaginal des microfilaires.

Par ailleurs, des localisations atypiques desmicrofilaires de *M. perstans* ont été mentionnées par certains auteurs dans l’abcès d’une glande salivaire par Lourdes et al [14]. Aussi, la localisation atypique en position intra oculaire a été notifiée par Cohen et al [13], et celle au niveau de la conjonctive par Baird et al [19].

Aussi, la localisation au niveau de la muqueuse cervico-vaginale est très atypique et exceptionnelle; deux cas ont été décrits, l’un par Sharma et al [17] en 1971, l’autre par Punia et al [20] en 2005. Les microfilaires de *M. perstans* en effet, circulent dans le sang tandis que les vers adultes sont dans les séreuses [1]. Ces localisations atypiques existent parfois en l’absence de circulation de microfilaires dans le sang.

Sur le plan thérapeutique, *M. perstans* est peu sensible aux microfilaricides classiques [8, 10]. Des données contradictoires existent quant au bénéfice de la diéthylcarbamazine [21]. Le mebendazole réduit la densité sanguine en microfilaires mais après des traitements au long court [22]. L’albendazole, comme l’ivermectine, ne semblent pas non plus efficaces quand ils sont administrés en prise unique [10]. C’est la raison pour laquelle la patiente a reçu une bithérapie microfilaricide associant albendazole et ivermectine. Cette association réduit considérablement la concentration en microfilaires dans le sang [10]. Chez notre patiente, l’évolution a été favorable à 3 mois.

## Conclusion

Nous rapportons un troisième cas de la microfilaire M perstans sur un frottis cervico-vaginal. La découverte fortuite dans les frottis cervicaux – vaginaux de microfilaires témoigne d’une filariose en général infra clinique, sans pathologie associée du tractus génital féminin. La conduite thérapeutique est celle de toute filariose en pays endémique, devant tenir compte, entre autre, de la ré infestation dans la plupart des cas.

La première mise en évidence de la localisation vaginale des microfilaires de M perstans dans notre hôpital, suscite l’intérêt d’une recherche de microfilaires de *M. perstans* sur tous les frottis des femmes acceptant un dépistage volontaire du cancer du col de l’utérus dans le service de gynécologie-obstétrique, afin de déterminer la fréquence de cette localisation.
